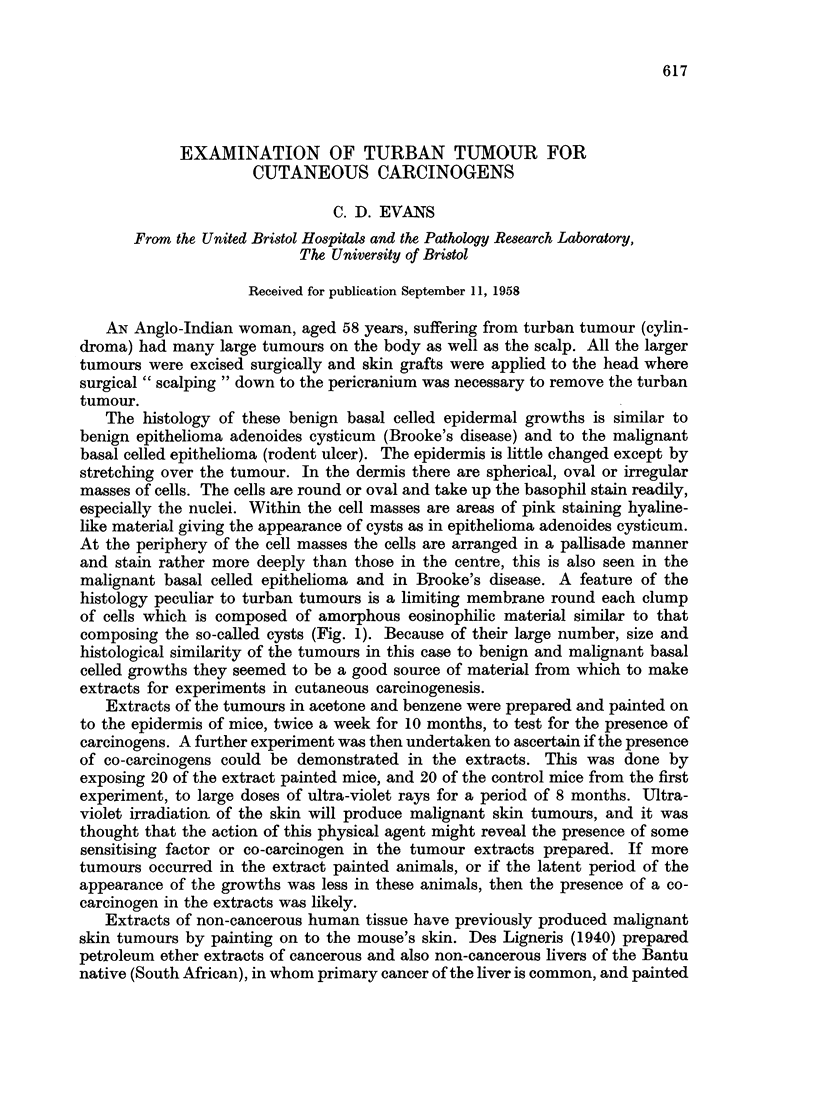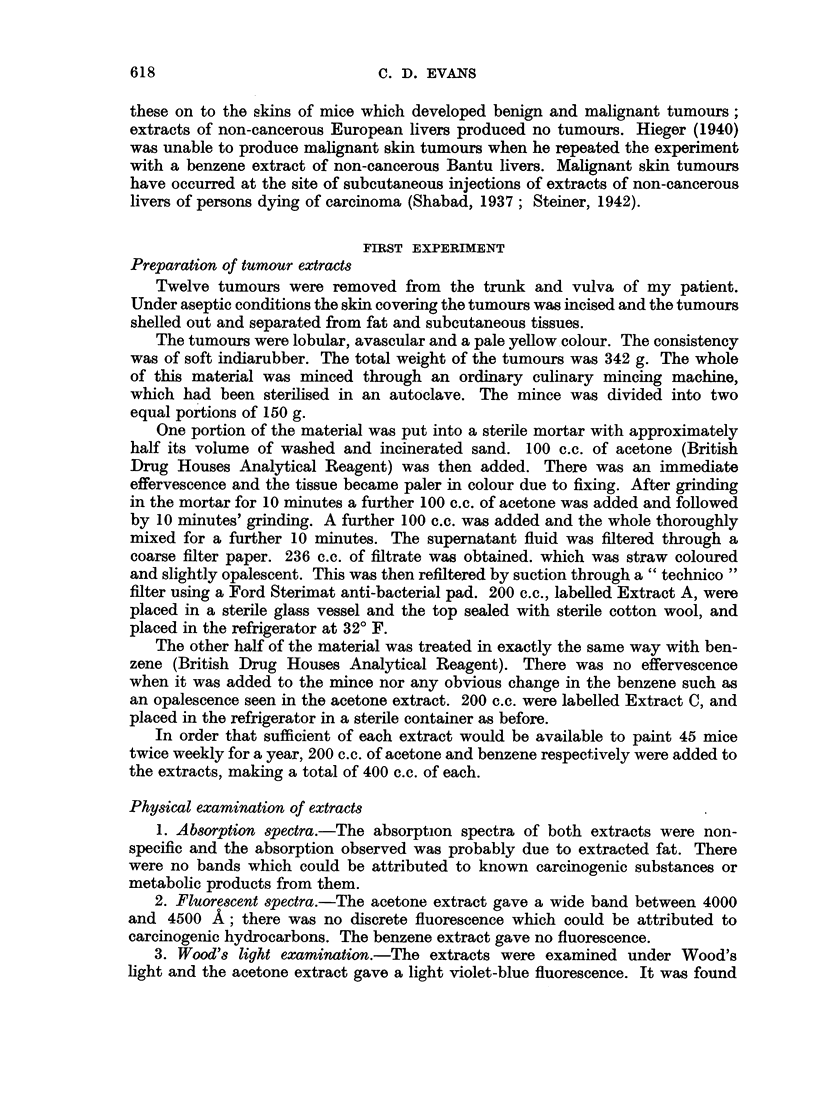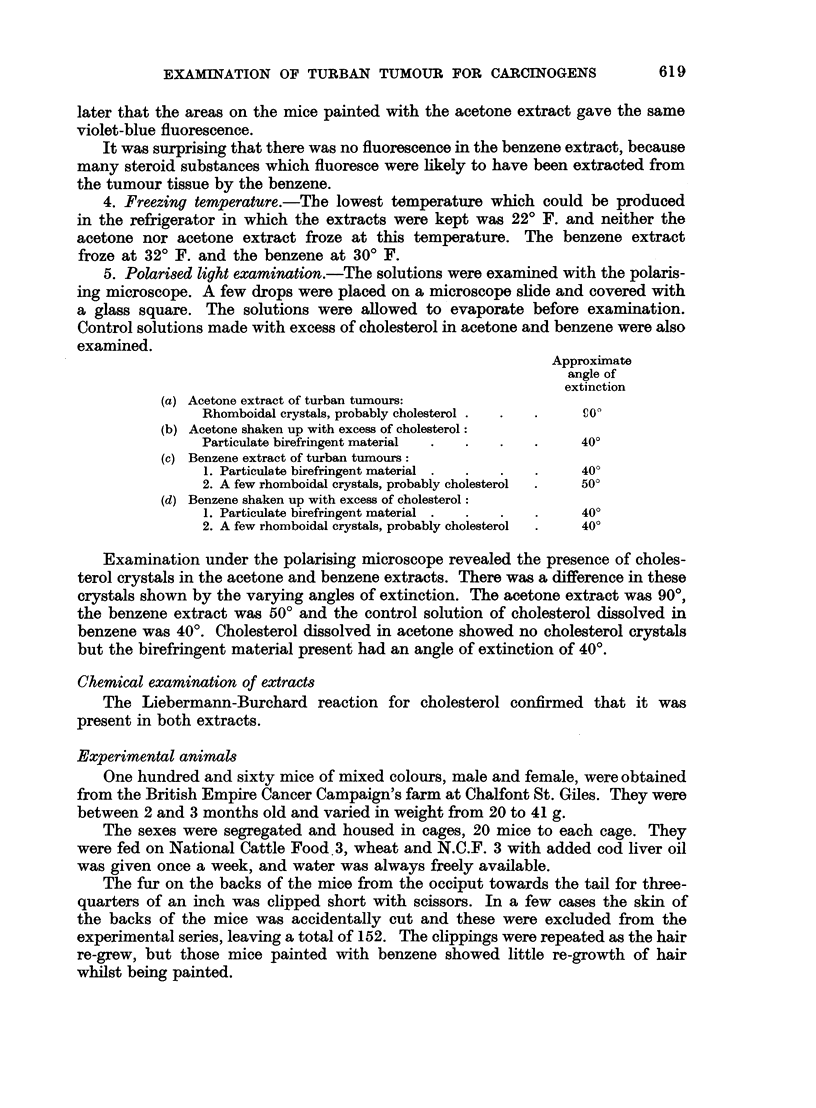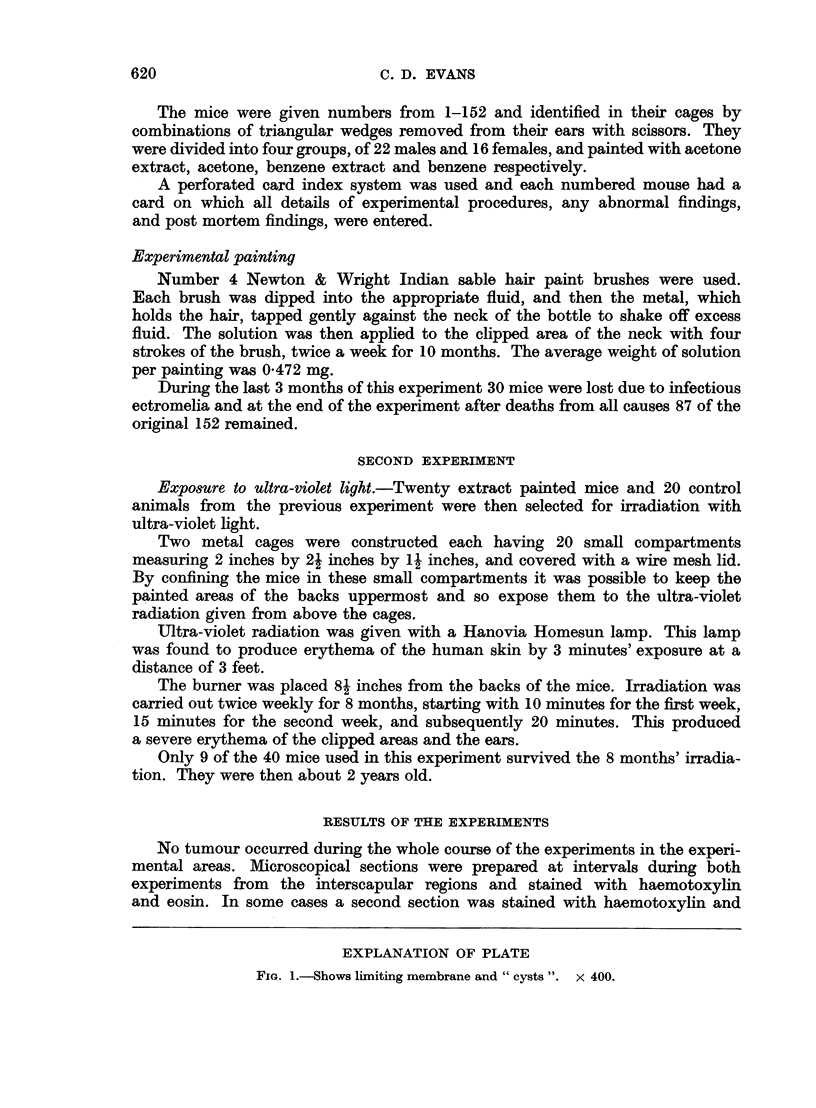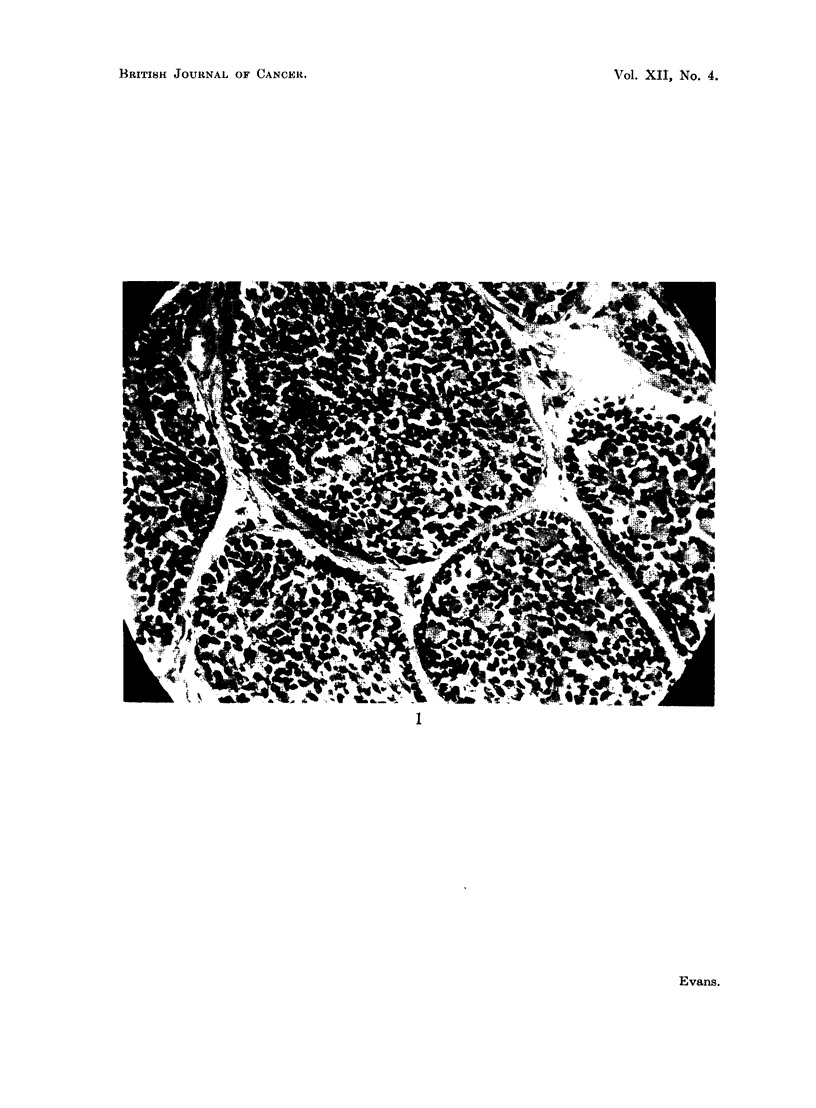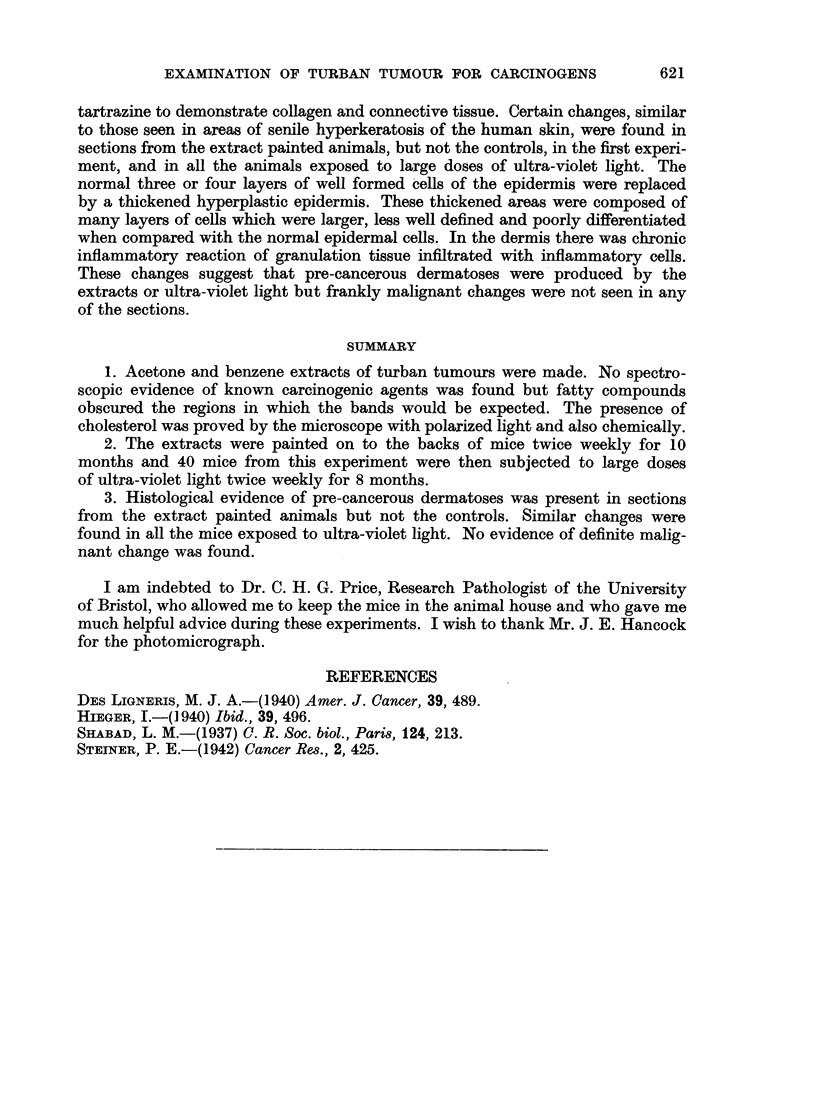# Examination of Turban Tumour for Cutaneous Carcinogens

**DOI:** 10.1038/bjc.1958.69

**Published:** 1958-12

**Authors:** C. D. Evans

## Abstract

**Images:**


### EXAMINATION OF TURBAN TUMOUR FOR CUTANEOUS CARCINOGENS

C. D. EVANS

*From the United Bristol Hospitals and the Pathology Research Laboratory, The University of Bristol*

Received for publication September 11, 1958

An Anglo-Indian woman, aged 58 years, suffering from turban tumour (cylindroma) had many large tumours on the body as well as the scalp. All the larger tumours were excised surgically and skin grafts were applied to the head where surgical " scalping " down to the pericranium was necessary to remove the turban tumour.

The histology of these benign basal celled epidermal growths is similar to benign epithelioma adenoides cysticum (Brooke's disease) and to the malignant basal celled epithelioma (rodent ulcer). The epidermis is little changed except by stretching over the tumour. In the dermis there are spherical, oval or irregular masses of cells. The cells are round or oval and take up the basophil stain readily, especially the nuclei. Within the cell masses are areas of pink staining hyaline-like material giving the appearance of cysts as in epithelioma adenoides cysticum. At the periphery of the cell masses the cells are arranged in a pallisade manner and stain rather more deeply than those in the centre, this is also seen in the malignant basal celled epithelioma and in Brooke's disease. A feature of the histology peculiar to turban tumours is a limiting membrane round each clump of cells which is composed of amorphous eosinophilic material similar to that composing the so-called cysts (Fig. 1). Because of their large number, size and histological similarity of the tumours in this case to benign and malignant basal celled growths they seemed to be a good source of material from which to make extracts for experiments in cutaneous carcinogenesis.

Extracts of the tumours in acetone and benzene were prepared and painted on to the epidermis of mice, twice a week for 10 months, to test for the presence of carcinogens. A further experiment was then undertaken to ascertain if the presence of co-carcinogens could be demonstrated in the extracts. This was done by exposing 20 of the extract painted mice, and 20 of the control mice from the first experiment, to large doses of ultra-violet rays for a period of 8 months. Ultra-violet irradiation of the skin will produce malignant skin tumours, and it was thought that the action of this physical agent might reveal the presence of some sensitising factor or co-carcinogen in the tumour extracts prepared. If more tumours occurred in the extract painted animals, or if the latent period of the appearance of the growths was less in these animals, then the presence of a co-carcinogen in the extracts was likely.

Extracts of non-cancerous human tissue have previously produced malignant skin tumours by painting on to the mouse's skin. Des Ligneris (1940) prepared petroleum ether extracts of cancerous and also non-cancerous livers of the Bantu native (South African), in whom primary cancer of the liver is common, and painted these on to the skins of mice which developed benign and malignant tumours; extracts of non-cancerous European livers produced no tumours. Hieger (1940) was unable to produce malignant skin tumours when he repeated the experiment with a benzene extract of non-cancerous Bantu livers. Malignant skin tumours have occurred at the site of subcutaneous injections of extracts of non-cancerous livers of persons dying of carcinoma (Shabad, 1937; Steiner, 1942).

### FIRST EXPERIMENT

*Preparation of tumour extracts*

Twelve tumours were removed from the trunk and vulva of my patient. Under aseptic conditions the skin covering the tumours was incised and the tumours shelled out and separated from fat and subcutaneous tissues.

The tumours were lobular, avascular and a pale yellow colour. The consistency was of soft indiarubber. The total weight of the tumours was 342 g. The whole of this material was minced through an ordinary culinary mincing machine, which had been sterilised in an autoclave. The mince was divided into two equal portions of 150 g.

One portion of the material was put into a sterile mortar with approximately half its volume of washed and incinerated sand. 100 c.c. of acetone (British Drug Houses Analytical Reagent) was then added. There was an immediate effervescence and the tissue became paler in colour due to fixing. After grinding in the mortar for 10 minutes a further 100 c.c. of acetone was added and followed by 10 minutes' grinding. A further 100 c.c. was added and the whole thoroughly mixed for a further 10 minutes. The supernatant fluid was filtered through a coarse filter paper. 236 c.c. of filtrate was obtained. which was straw coloured and slightly opalescent. This was then refiltered by suction through a " technico " filter using a Ford Sterimat anti-bacterial pad. 200 c.c., labelled Extract A, were placed in a sterile glass vessel and the top sealed with sterile cotton wool, and placed in the refrigerator at 32° F.

The other half of the material was treated in exactly the same way with benzene (British Drug Houses Analytical Reagent). There was no effervescence when it was added to the mince nor any obvious change in the benzene such as an opalescence seen in the acetone extract. 200 c.c. were labelled Extract C, and placed in the refrigerator in a sterile container as before.

In order that sufficient of each extract would be available to paint 45 mice twice weekly for a year, 200 c.c. of acetone and benzene respectively were added to the extracts, making a total of 400 c.c. of each.

*Physical examination of extracts*

1. *Absorption spectra.*—The absorption spectra of both extracts were non-specific and the absorption observed was probably due to extracted fat. There were no bands which could be attributed to known carcinogenic substances or metabolic products from them.

2. *Fluorescent spectra.*—The acetone extract gave a wide band between 4000 and 4500 Å; there was no discrete fluorescence which could be attributed to carcinogenic hydrocarbons. The benzene extract gave no fluorescence.

3. *Wood's light examination.*—The extracts were examined under Wood's light and the acetone extract gave a light violet-blue fluorescence. It was found later that the areas on the mice painted with the acetone extract gave the same violet-blue fluorescence.

It was surprising that there was no fluorescence in the benzene extract, because many steroid substances which fluoresce were likely to have been extracted from the tumour tissue by the benzene.

4. *Freezing temperature.*—The lowest temperature which could be produced in the refrigerator in which the extracts were kept was 22° F. and neither the acetone nor acetone extract froze at this temperature. The benzene extract froze at 32° F. and the benzene at 30° F.

5. *Polarised light examination.*—The solutions were examined with the polarising microscope. A few drops were placed on a microscope slide and covered with a glass square. The solutions were allowed to evaporate before examination. Control solutions made with excess of cholesterol in acetone and benzene were also examined.

| | | Approximate angle of extinction |
|---|---|---|
| (*a*) | Acetone extract of turban tumours: | |
| | Rhomboidal crystals, probably cholesterol | 90° |
| (b) | Acetone shaken up with excess of cholesterol: | |
| | Particulate birefringent material | 40° |
| (*c*) | Benzene extract of turban tumours: | |
| | 1. Particulate birefringent material | 40° |
| | 2. A few rhomboidal crystals, probably cholesterol | 50° |
| (*d*) | Benzene shaken up with excess of cholesterol: | |
| | 1. Particulate birefringent material | 40° |
| | 2. A few rhomboidal crystals, probably cholesterol | 40° |

Examination under the polarising microscope revealed the presence of cholesterol crystals in the acetone and benzene extracts. There was a difference in these crystals shown by the varying angles of extinction. The acetone extract was 90°, the benzene extract was 50° and the control solution of cholesterol dissolved in benzene was 40°. Cholesterol dissolved in acetone showed no cholesterol crystals but the birefringent material present had an angle of extinction of 40°.

*Chemical examination of extracts*

The Liebermann-Burchard reaction for cholesterol confirmed that it was present in both extracts.

*Experimental animals*

One hundred and sixty mice of mixed colours, male and female, were obtained from the British Empire Cancer Campaign's farm at Chalfont St. Giles. They were between 2 and 3 months old and varied in weight from 20 to 41 g.

The sexes were segregated and housed in cages, 20 mice to each cage. They were fed on National Cattle Food 3, wheat and N.C.F. 3 with added cod liver oil was given once a week, and water was always freely available.

The fur on the backs of the mice from the occiput towards the tail for three-quarters of an inch was clipped short with scissors. In a few cases the skin of the backs of the mice was accidentally cut and these were excluded from the experimental series, leaving a total of 152. The clippings were repeated as the hair re-grew, but those mice painted with benzene showed little re-growth of hair whilst being painted.

The mice were given numbers from 1–152 and identified in their cages by combinations of triangular wedges removed from their ears with scissors. They were divided into four groups, of 22 males and 16 females, and painted with acetone extract, acetone, benzene extract and benzene respectively.

A perforated card index system was used and each numbered mouse had a card on which all details of experimental procedures, any abnormal findings, and post mortem findings, were entered.

*Experimental painting*

Number 4 Newton & Wright Indian sable hair paint brushes were used. Each brush was dipped into the appropriate fluid, and then the metal, which holds the hair, tapped gently against the neck of the bottle to shake off excess fluid. The solution was then applied to the clipped area of the neck with four strokes of the brush, twice a week for 10 months. The average weight of solution per painting was 0·472 mg.

During the last 3 months of this experiment 30 mice were lost due to infectious ectromelia and at the end of the experiment after deaths from all causes 87 of the original 152 remained.

### SECOND EXPERIMENT

*Exposure to ultra-violet light.*—Twenty extract painted mice and 20 control animals from the previous experiment were then selected for irradiation with ultra-violet light.

Two metal cages were constructed each having 20 small compartments measuring 2 inches by $2\frac{1}{2}$ inches by $1\frac{1}{2}$ inches, and covered with a wire mesh lid. By confining the mice in these small compartments it was possible to keep the painted areas of the backs uppermost and so expose them to the ultra-violet radiation given from above the cages.

Ultra-violet radiation was given with a Hanovia Homesun lamp. This lamp was found to produce erythema of the human skin by 3 minutes' exposure at a distance of 3 feet.

The burner was placed $8\frac{1}{2}$ inches from the backs of the mice. Irradiation was carried out twice weekly for 8 months, starting with 10 minutes for the first week, 15 minutes for the second week, and subsequently 20 minutes. This produced a severe erythema of the clipped areas and the ears.

Only 9 of the 40 mice used in this experiment survived the 8 months' irradiation. They were then about 2 years old.

### RESULTS OF THE EXPERIMENTS

No tumour occurred during the whole course of the experiments in the experimental areas. Microscopical sections were prepared at intervals during both experiments from the interscapular regions and stained with haemotoxylin and eosin. In some cases a second section was stained with haemotoxylin and

---

EXPLANATION OF PLATE

FIG. 1.—Shows limiting membrane and " cysts ". × 400.

1

tartrazine to demonstrate collagen and connective tissue. Certain changes, similar to those seen in areas of senile hyperkeratosis of the human skin, were found in sections from the extract painted animals, but not the controls, in the first experiment, and in all the animals exposed to large doses of ultra-violet light. The normal three or four layers of well formed cells of the epidermis were replaced by a thickened hyperplastic epidermis. These thickened areas were composed of many layers of cells which were larger, less well defined and poorly differentiated when compared with the normal epidermal cells. In the dermis there was chronic inflammatory reaction of granulation tissue infiltrated with inflammatory cells. These changes suggest that pre-cancerous dermatoses were produced by the extracts or ultra-violet light but frankly malignant changes were not seen in any of the sections.

SUMMARY

1. Acetone and benzene extracts of turban tumours were made. No spectroscopic evidence of known carcinogenic agents was found but fatty compounds obscured the regions in which the bands would be expected. The presence of cholesterol was proved by the microscope with polarized light and also chemically.

2. The extracts were painted on to the backs of mice twice weekly for 10 months and 40 mice from this experiment were then subjected to large doses of ultra-violet light twice weekly for 8 months.

3. Histological evidence of pre-cancerous dermatoses was present in sections from the extract painted animals but not the controls. Similar changes were found in all the mice exposed to ultra-violet light. No evidence of definite malignant change was found.

I am indebted to Dr. C. H. G. Price, Research Pathologist of the University of Bristol, who allowed me to keep the mice in the animal house and who gave me much helpful advice during these experiments. I wish to thank Mr. J. E. Hancock for the photomicrograph.

### REFERENCES

Des Ligneris, M. J. A.—(1940) *Amer. J. Cancer*, **39**, 489.
Hieger, I.—(1940) *Ibid.*, **39**, 496.
Shabad, L. M.—(1937) *C. R. Soc. biol., Paris*, **124**, 213.
Steiner, P. E.—(1942) *Cancer Res.*, **2**, 425.